# Assessment of Choroidal Vascularity and Choriocapillaris Blood Perfusion After Accommodation in Myopia, Emmetropia, and Hyperopia Groups Among Children

**DOI:** 10.3389/fphys.2022.854240

**Published:** 2022-03-17

**Authors:** Xuejiao Chang, Mu Li, Liang Lv, Xiaoqin Yan, Ying Liu, Mengxia Zhu, Junming Wang, Ping Wang, Yan Xiang

**Affiliations:** ^1^Department of Ophthalmology, Tongji Hospital, Tongji Medical College, Huazhong University of Science and Technology, Wuhan, China; ^2^Department of Ophthalmology, Union Hospital, Tongji Medical College, Huazhong University of Science and Technology, Wuhan, China; ^3^Department of Ophthalmology, Hankou Aier Eye Hospital, Wuhan, China

**Keywords:** myopia, accommodation, choroidal vascularity, choriocapillaris, optical coherence tomography

## Abstract

**Purpose:**

To investigate choroidal vascularity (CV) and choriocapillaris blood perfusion before and after accommodation in myopia, emmetropia, and hyperopia groups among children.

**Methods:**

This study included 39 myopic eyes from 22 subjects, 17 emmetropic eyes from 11 subjects, and 18 hyperopic eyes from 10 subjects. All subjects were children. Choroidal thickness (CT) and CV, including total choroidal area (TCA), luminal area (LA), and stromal area (SA) were measured using swept-source optical coherence tomography (SS-OCT). Choriocapillaris luminal area (CLA) was measured using SS-OCT-angiography before and after accommodation (near reading with an additional −3 diopter lens).

**Results:**

For baseline results, except horizontal CV (showing no significant differences between myopia and emmetropia groups), both horizontal and vertical CT and CV were significantly smaller in the myopia group than in the emmetropia or hyperopia groups. In terms of CLA, no significant differences were observed among the myopia, emmetropia, and hyperopia groups. In addition, only myopic eyes showed significant decreases in CT and CV, whereas most CT and CV of emmetropic and hyperopic eyes showed non-significant decreases after accommodation. Furthermore, accommodation induced no significant changes in CLA in the myopia, emmetropia, or hyperopia groups.

**Conclusion:**

Myopia had thinner baseline choroid and lower baseline choroidal blood perfusion. Furthermore, myopic eyes were more prone to choroidal thinning and blood perfusion decreases after accommodation.

## Introduction

Myopia is one of the leading ophthalmologic disorders worldwide (Baird et al., 2020). By 2050, the global prevalence of myopia would approach nearly 50%, and the prevalence of high myopia would be 9.8% (Holden et al., 2016). The annual incidence rate of myopia in school-aged children could be as high as 20–30% (Wang et al., 2018). There is currently an epidemic of myopia, especially in East Asia (Dayan et al., 2005; Dolgin, 2015; Lin et al., 2018; Wang et al., 2018; Spillmann, 2020). Myopia is characterized by an excessive increase in axial length (AL) and is associated with various complications, including retinal detachment, myopic maculopathy, and glaucoma (Verhoeven et al., 2015). All of these complications could lead to irreversible visual impairment; thus, myopia is an important factor in low vision worldwide.

Although the exact etiology of myopia remains unclear, it is suggested to have both genetic and environmental factors (Woodman et al., 2011). Near work has been reported to be an important environmental component contributing to the development of myopia (Goss, 2000; Ip et al., 2008).

In addition, in recent years, a close association between changes in choroidal thickness (CT) and ocular growth has been reported, suggesting that thinning in the choroid accompanies the development of myopia (Read et al., 2013, 2015; Wei et al., 2013; Xiong et al., 2020). The thinning of the choroid occurs early during myopia development and is accompanied by accelerated ocular growth. In contrast, a slower ocular growth was observed with thicker choroid (Wallman et al., 1995; Wildsoet and Wallman, 1995; Hung et al., 2000; Zhu et al., 2005; Read et al., 2015; Zhang et al., 2019; Xiong et al., 2020). Besides CT, the choroidal vasculature and perfusion were also reported to be decreased in myopia and could be a contributing factor to the development of myopia (Mo et al., 2017; Zhang et al., 2019; Wu et al., 2021a,b). Thus, choroid may also play an important role in the development of myopia.

Swept-source optical coherence tomography (SS-OCT) in combination with OCT angiography (OCTA) allows us to obtain choroidal information, such as CT, choroidal vascularity (CV), and choriocapillaris luminal area (CLA) comprehensively and simultaneously; it has been widely used for choroidal studies (Zhang et al., 2019; Wu et al., 2021a,b). To date, previous accommodation-related choroidal studies have been mostly conducted in adults (Woodman et al., 2012; Ghosh et al., 2014; Woodman-Pieterse et al., 2015; Yoon et al., 2021), and only few studies have investigated the effects of accommodation in the choroid of children, which is the main stage of myopia development (Cumberland et al., 2007; Morgan et al., 2012; Golebiewska et al., 2019). Furthermore, most previous accommodation-related choroidal studies only included emmetropia and myopia groups but not a hyperopia group. In this study, we recruited a wider range of refraction status, including hyperopia, emmetropia, and myopia. In this study, we aimed to compare the CT, CV, and CLA of children with myopia, emmetropia, and hyperopia, and to observe changes in CT, CV, and CLA of children with myopia, emmetropia, and hyperopia after accommodation using SS-OCT/OCTA. Considering that near reading is proven to be an important factor for the development of myopia (Goss, 2000; Ip et al., 2008), we used near reading with an additional −3 Diopter (D) lens, instead of only using a negative lens, as accommodation stimuli.

## Materials and Methods

The study was approved by the ethics committee of Tongji Hospital, Tongji Medical College, Huazhong University of Science and Technology and adhered to the tenets of the Declaration of Helsinki. The guardians of the enrolled subjects signed written informed consent before participation.

### Subjects

Thirty-eight children (age range 6–12 years) were recruited for this prospective study. Before participation, all subjects underwent comprehensive ophthalmic examinations, including best-corrected visual acuity (BCVA) assessment based on the Snellen chart, intraocular pressure measurement, cycloplegic optometry, comprehensive optometry, amplitude of accommodation measurement, AL measurement, fundus photography, and slit-lamp examination. All subjects had a BCVA ≥1.0, and the amplitudes of accommodation were ≥8 D. Subjects with ocular diseases other than refractive error, with a history of ocular trauma or surgery, wearing orthokeratology lenses, or experiencing systemic disease were excluded from recruitment.

Both eyes of each subject were included. The eyes were divided into three groups according to the cycloplegic optometry results. Myopia was defined as a spherical equivalent (SE) ≤ −0.75 D (40 myopic eyes from 22 subjects), emmetropia as −0.75 D < SE < +1.00 D (17 emmetropic eyes from 11 subjects), and hyperopia as +1.00 D ≤ SE < +5.00 D (19 hyperopic eyes from 10 subjects; Williams et al., 2015). One myopic eye and one hyperopic eye were excluded from further image and data analysis due to the low image quality. All subjects were requested to abstain from caffeine for 3 days and from any food or beverage for at least 30 min before study participation (Li et al., 2018).

### SS-OCT/OCTA Image Acquisition Before and After Accommodation (33-cm Near Reading With Additional −3 D Lens)

All subjects were instructed to watch an electronic display at a distance of 5 m for 20 min with their full-distance spectacle corrections (Wu et al., 2021b). Subsequently, the subjects underwent SS-OCT/OCTA examination to obtain baseline choroidal images. After that, accommodation was performed on the study subjects. Based on the results of cycloplegic and comprehensive optometry, an additional −3 D lens was added to each study subject, and the subject was instructed to read a children’s book at a close distance of 33 cm for 30 min. The subject should keep a clear focus on the text paragraphs of the book during the near reading with an additional −3 D lens. To ensure this, the subject was required to read the text paragraphs loudly and accurately. All subjects read the book under the same light brightness (500 lux) and with the same reading material. SS-OCT/OCTA examination was performed immediately after 30 min of accommodation (near reading with an additional −3 D lens) to obtain post-reading choroidal images. Follow-up mode was used in advance to ensure that the scans before and after accommodation were in the same position and that the scans could be performed quickly after accommodation. The post-accommodation OCT/OCTA measurements were completed within 30–60 s. All scans were conducted between 14:30 and 17:30 each day to minimize the potential effect of diurnal variation of the choroid (Tan et al., 2012; Li et al., 2017a,b).

### SS-OCT/OCTA Image Analysis

The SS-OCT/OCTA system (VG200S; SVision Imaging, Henan, China) used a 1,050-nm wavelength scanning laser. A confocal scanning laser ophthalmoscope was integrated for eye-tracking to eliminate eye-motion artifacts. The main scan parameters were as follows: axial resolution: 5 μm, lateral resolution: 13 μm, scan depth: 3 mm (Wu et al., 2021b).

**Figure 1:**
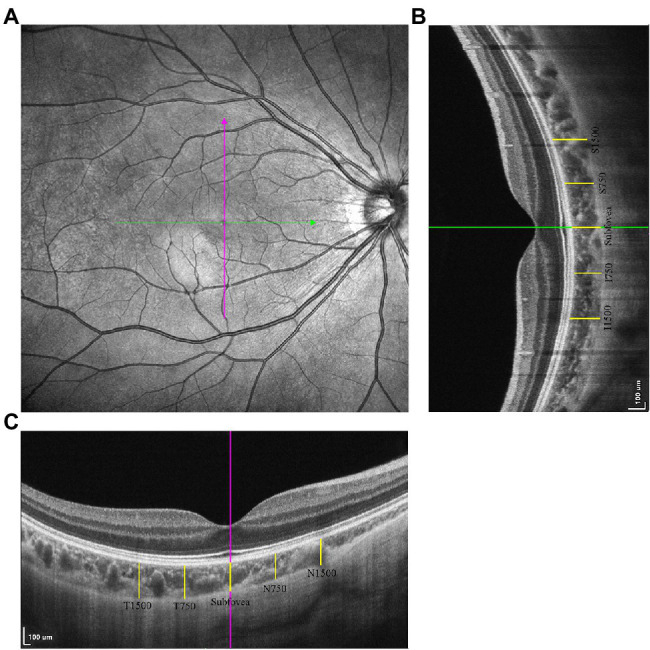
The measurements of choroidal thickness (CT). **(A)** The horizontal and vertical scan line centering on the fovea. **(B)** The choroidal thickness measurements in vertical scan. **(C)** The choroidal thickness measurements in horizontal scan.

Structural OCT was conducted with horizontal and vertical scan lines centered on the fovea with a length of 6 mm (Figure 1A). Sixteen B-scans were contained in each scan line and were automatically averaged to enhance the signal-to-noise ratio (Alonso-Caneiro et al., 2011). The choroid in the SS-OCT images was defined as the area from the retinal pigment epithelium (RPE)–Bruch’s membrane complex to the choroid–sclera interface (Li et al., 2017a,b; Wu et al., 2021b).

Choroidal thickness was defined as the distance between the RPE–Bruch’s membrane complex and the choroid–sclera interface (Noce et al., 2020), measured using ImageJ software (Figures 1B,C). The measurement locations were the fovea in both the horizontal and vertical scans, 750/1,500 μm nasal/temporal from the fovea on horizontal scans (N750, N1500, T750, T1500), and 750/1,500 μm superior/inferior from the fovea in vertical scans (S750, S1500, I750, I1500).

**Figure 2:**
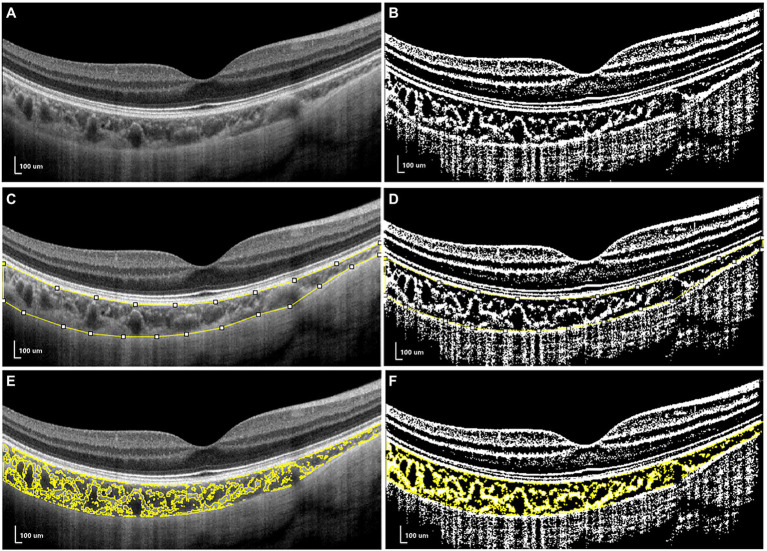
The measurements of choroidal vascularity (CV). **(A,C,E)** the structural optical coherence tomography (OCT) image. **(B,D,F)** the binarized OCT image.

For CV analysis, image binarization was conducted using ImageJ. The total choroidal area (TCA) was selected from the outer boundary of the RPE–Bruch’s membrane layer to the choroid–sclera interface, with a 3-mm width in both directions from the fovea (total length of TCA, 6 mm; Figure 2A). TCA was selected as a region of interest (ROI) using a polygon selection tool and added to the ROI manager. The image was converted to an 8-bit image to allow the application of the Niblack auto local threshold tool. The binarized image was re-converted to an RGB image, and the luminal area (LA) was highlighted using a color threshold tool and further added to the ROI manager. To determine the LA within the TCA polygon, both areas from the ROI manager were selected and merged using the “AND” operation. This third area was then added to the ROI manager (Figures 2E,F). The stromal area (SA) was obtained after the LA was subtracted from the TCA (Tan and Agrawal, 2015; Agrawal et al., 2016; Lee et al., 2020; Figure 2).

**Figure 3:**
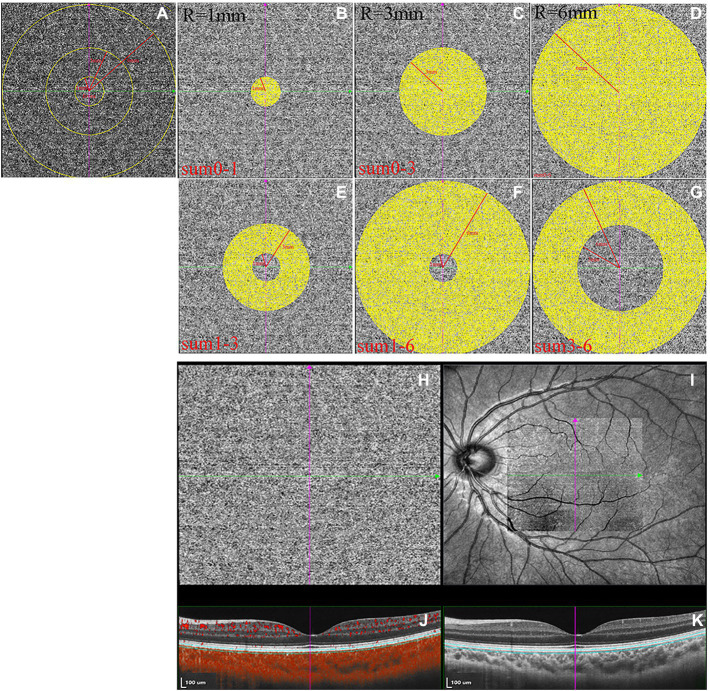
The measurements of choriocapillaris luminal area (CLA). **(A–G)** The measurement ranges of CLA. **(H)** Magnified en face optical coherence tomography angiography (OCTA) choriocapillaris image. **(I)** OCTA scan region of 6 × 6 mm centering on fovea. **(J)** Structural optical coherence tomography (OCT) image combined with flow OCT image with choriocapillaris segmentation lines. **(K)** Structural OCT image.

OCTA images were collected using the SVision SS-OCTA algorithm. The image acquisition was a raster scan of 512 B-scans covering an area of 6 × 6 mm centered on the fovea. Each B-scan was repeated four times, and the results were averaged (Wu et al., 2021b). The en face angiograms of the choriocapillaris slab (a layer starting at the basal border of the RPE–Bruch’s membrane complex and ending at approximately 20 μm beneath the RPE–Bruch’s membrane complex) were evaluated, and projection artifacts from retinal vessels were removed using the algorithm. CLA was defined as areas with flow signals that were detectable by the threshold binarization algorithm (Figures 3H–K; Zhang et al., 2018; Wu et al., 2021b). Based on the Early Treatment Diabetic Retinopathy Study grid, the macular region was divided into areas consisting of three concentric rings with diameters of 1, 3, and 6 mm centering on the fovea (Figure 3A). This grid was used in this study for choriocapillaris angiography analysis. The measurement locations were as follows: the area between the fovea and 1 mm from the fovea (0-1sum), area between the fovea and 3 mm from the fovea (0-3sum), area between the fovea and 6 mm from the fovea (0–6sum), area between 1 mm from the fovea and 3 mm from the fovea (1-3sum), area between 1 mm from the fovea and 6 mm from the fovea (1-6sum), and area between 3 mm from the fovea and 6 mm from the fovea (3-6sum, Figures 3B–G). En face angiograms were exported for analysis, and brightness and contrast of the images were not adjusted, ensuring that the images were manipulated in the native form. All image analyses were performed using ImageJ. We used the “round selection tool” to create concentric rings with diameters of 1, 3, and 6 mm centered on the fovea and added them to the ROI manager. We then merged the three circles using the “AND” operation to form three rings (Figure 3A). The image was converted to an 8-bit image to allow for the application of the Phansalkar auto local threshold tool. The binarized image was re-converted to an RGB image, and the CLA was highlighted using a color threshold tool and further added to the ROI manager. To determine the CLA within the en face choriocapillaris, all areas from the ROI manager were selected and merged using the “AND” operation.

Among the 76 eyes of 38 study subjects, two eyes were excluded from further analysis due to the low image quality. Thus, we finally recruited 74 eyes from 38 participants (39 myopic eyes from 22 subjects, 17 emmetropic eyes from 11 subjects, 18 hyperopic eyes from 10 subjects) for data measurement and analysis. All the measurements were masked to the subject information. To ensure the accuracy of measurements, 30 eyes (18 myopic eyes, six emmetropic eyes and six hyperopic eyes) were randomly selected, and CT, CV, and CLA were re-measured by the trained examiners (XC). The intraclass correlation coefficient values of the two independent measurements of CT were range from 0.857 to 0.927, of CV were range from 0.882 to 0.947, and of CLA were range from 0.999 to 1.000.

### Statistical Analysis

All analyses were performed by SPSS software 21.0 (IBM Corp., Armonk, NY, United States). Data were shown as mean ± standard deviation where applicable. The age data distribution was tested with Shapiro–Wilk test and found to be Non-Gaussian. Thus, Kruskal–Wallis H test was used for the inter-group comparison of age. Chi-square statistic was used for the inter-group comparison of sex. Generalized estimating equations (GEEs), which take the correlation of measurements of two eyes of one subject into account, was used for the inter-group comparison of axial length, spherical equivalent, baseline CT, baseline CV, baseline CLA and for the pre- and post-accommodation comparison of CT, CV and CLA. The univariate linear regression of associations of baseline choroidal parameters with age, sex, axial length was also performed using GEEs, assuming linear model.

## Results

As shown in Table 1, there were no significant age or sex differences among the myopia, emmetropia, and hyperopia groups (all *p* > 0.05). In terms of AL and SE, the three enrolled groups showed significant differences between each other, with the myopia group having the longest AL and lowest SE and the hyperopia group having the shortest AL and highest SE (all *p* < 0.05).

**Table 1:** Subject characteristics.

	Myopia	Emmetropia	Hyperopia	*p1*	*p2*	*p3*
Age (years)	8.55 ± 1.63	8.18 ± 2.44	7.90 ± 1.85	0.313	0.196	0.914
Sex (male/female)	10/12	5/6	4/6	1.000	0.773	0.801
Axial length (mm)	24.30 ± 0.95	22.53 ± 0.90	21.61 ± 0.81	<0.001^*^	<0.001^*^	0.018^*^
Spherical equivalent (D)	−2.21 ± 1.26	0.35 ± 0.46	3.27 ± 1.28	<0.001^*^	<0.001^*^	<0.001^*^

p1: Comparison between myopia and emmetropia; p2: Comparison between myopia and hyperopia; p3: Comparison between emmetropia and hyperopia. Kruskal–Wallis H test for the inter-group comparison of age; Chi-square statistic for the inter-group comparison of sex; GEEs for the inter-group comparison of axial length and spherical equivalent.

* Significance of difference.

### Comparisons of Baseline CT, CV, and CLA Among the Myopia, Emmetropia, and Hyperopia Groups

The CT of the myopia group was significantly thinner than those of the emmetropia and hyperopia groups in all measured locations (subfovea, 750/1,500 μm nasal/temporal/superior/inferior from fovea; all *p* < 0.05). In addition, the CT of the emmetropia group was significantly thinner than that of the hyperopia group at 750/1,500 μm nasal/superior from the fovea (all *p* < 0.05; Table 2).

**Table 2:** Baseline choroidal thickness (CT) comparisons among myopia, emmetropia, and hyperopia groups.

	Myopia	Emmetropia	Hyperopia	*p1*	*p2*	*p3*
*Horizontal*						
Subfovea (μm)	247.03 ± 41.64	315.38 ± 74.97	365.76 ± 83.35	0.004^*^	<0.001^*^	0.115
N750 (μm)	222.16 ± 6.44	285.76 ± 6.09	345.96 ± 1.70	0.002^*^	<0.001^*^	0.040^*^
N1500 (μm)	192.47 ± 6.24	243.38 ± 8.46	323.89 ± 79.27	0.018^*^	<0.001^*^	0.006^*^
T750 (μm)	257.18 ± 42.91	320.12 ± 69.63	358.23 ± 75.58	0.003^*^	<0.001^*^	0.174
T1500 (μm)	267.82 ± 43.81	325.67 ± 72.44	351.55 ± 73.81	0.009^*^	<0.001^*^	0.360
Mean (μm)	237.33 ± 36.90	298.06 ± 66.38	349.08 ± 74.12	0.004^*^	<0.001^*^	0.068
*Vertical*						
Subfovea (μm)	247.55 ± 39.81	312.63 ± 63.97	361.25 ± 87.68	0.001^*^	<0.001^*^	0.085
S750 (μm)	236.62 ± 38.97	309.82 ± 59.10	365.75 ± 80.15	<0.001^*^	<0.001^*^	0.036^*^
S1500 (μm)	243.64 ± 35.24	305.84 ± 61.17	369.16 ± 77.93	0.002^*^	<0.001^*^	0.017^*^
I750 (μm)	244.26 ± 39.13	309.16 ± 66.21	334.89 ± 85.36	0.002^*^	<0.001^*^	0.362
I1500 (μm)	237.70 ± 40.10	304.00 ± 68.22	329.87 ± 75.25	0.002^*^	<0.001^*^	0.335
Mean (μm)	241.96 ± 34.81	308.29 ± 61.73	352.18 ± 78.73	0.001^*^	<0.001^*^	0.101

p1: Comparison between myopia and emmetropia; p2: Comparison between myopia and hyperopia; p3: Comparison between emmetropia and hyperopia. GEEs for the inter-group comparison. Age and sex have been adjusted. N, nasal; T, temporal; S, superior; I, inferior.

* Significance of difference.

For the horizontal scans of baseline CV, the LA, SA, and TCA were significantly smaller in the myopia group than in the hyperopia group (all *p* < 0.05). However, no such significant differences were found between the emmetropia and myopia/hyperopia groups (all *p* > 0.05). For the vertical scans of baseline CV, the LA, SA, and TCA were significantly smaller in the myopia group than in the emmetropia and hyperopia groups (all *p* < 0.05), whereas no such significant differences were found between the emmetropia and hyperopia groups (all *p* > 0.05; Table 3). In terms of baseline CLA, there were no significant differences among the myopia, emmetropia, and hyperopia groups in any measurement ranges (all *p* > 0.05; Table 4).

**Table 3:** Baseline choroidal vascularity (CV) comparisons among myopia, emmetropia and hyperopia groups.

	Myopia	Emmetropia	Hyperopia	*p1*	*p2*	*p3*
*Horizontal*						
Luminal area (LA, μm^2^)	1,015,977 ± 149,135	1,160,080 ± 261,866	1,292,542 ± 250,185	0.108	0.001^*^	0.200
Stromal area (SA, μm^2^)	542,845 ± 78,751	609,105 ± 147,207	689,878 ± 129,682	0.087	0.001^*^	0.225
Total choroidal area (TCA, μm^2^)	1,558,823 ± 214,840	1,769,185 ± 406,063	1,982,421 ± 368,901	0.166	<0.001^*^	0.170
*Vertical*						
Luminal area (LA, μm^2^)	1,108,748 ± 133,397	1,262,953 ± 223,965	1,333,644 ± 220,260	0.027^*^	0.001^*^	0.466
Stromal area (SA, μm^2^)	592,642 ± 73,458	669,611 ± 126,311	703,104 ± 142,895	0.031^*^	<0.001^*^	0.446
Total choroidal area (TCA, μm^2^)	1,701,391 ± 193,186	1,932,564 ± 340,377	2,036,749 ± 355,880	0.037^*^	0.005^*^	0.540

p1: Comparison between myopia and emmetropia; p2: Comparison between myopia and hyperopia; p3: Comparison between emmetropia and hyperopia. GEEs for the inter-group comparison. Age and sex have been adjusted.

* Significance of difference.

**Table 4:** Baseline choriocapillaris luminal area (CLA) comparisons among myopia, emmetropia, and hyperopia groups.

	Myopia	Emmetropia	Hyperopia	*p1*	*p2*	*p3*
0-1sum (mm^2^)	0.626 ± 0.036	0.611 ± 0.040	0.623 ± 0.032	0.118	0.665	0.379
0-3sum (mm^2^)	5.696 ± 0.354	5.587 ± 0.368	5.719 ± 0.308	0.216	0.978	0.343
0-6sum (mm^2^)	22.313 ± 1.334	22.095 ± 1.260	22.545 ± 1.177	0.407	0.789	0.361
1-3sum (mm^2^)	5.069 ± 0.319	4.976 ± 0.331	5.096 ± 0.279	0.233	0.986	0.342
1-6sum (mm^2^)	21.687 ± 1.299	21.483 ± 1.224	21.922 ± 1.148	0.421	0.774	0.361
3-6sum (mm^2^)	16.617 ± 0.990	16.507 ± 0.904	16.826 ± 0.877	0.511	0.709	0.372

p1: Comparison between myopia and emmetropia; p2: Comparison between myopia and hyperopia; p3: Comparison between emmetropia and hyperopia. GEEs for the inter-group comparison. Age and sex have been adjusted.

### Comparisons of CT, CV, and CLA Before and After Accommodation in the Myopia, Emmetropia, and Hyperopia Groups

**Figure 4:**
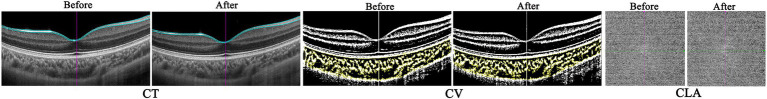
The representative optical coherence tomography (OCT) and OCT angiography (OCTA) images of one myopic eye before and after accommodation.

After accommodation, all measured CTs decreased significantly in the myopia group (all *p* < 0.001). In the emmetropia group, only the CT of the subfovea (in both horizontal and vertical scans), 750 μm superior from the fovea, and mean vertical CT showed a significant decrease after accommodation (all *p* < 0.05). In the hyperopia group, only horizontal subfoveal CT, mean horizontal CT, and CT of 750 μm nasal/superior from the fovea decreased significantly after accommodation (all *p* < 0.05; Table 5 and Figure 4).

**Table 5:** Comparisons of choroidal thickness (CT) before and after accommodation in myopia, emmetropia and hyperopia groups.

	Before reading	After reading	*p*
*Myopia*			
*Horizontal*			
Subfovea (μm)	247.03 ± 41.64	235.13 ± 39.53	<0.001^*^
N750 (μm)	222.16 ± 6.44	211.18 ± 37.46	<0.001^*^
N1500 (μm)	192.47 ± 6.24	180.01 ± 36.90	<0.001^*^
T750 (μm)	257.18 ± 42.91	246.31 ± 41.57	<0.001^*^
T1500 (μm)	267.82 ± 43.81	256.40 ± 42.30	<0.001^*^
Mean (μm)	237.33 ± 36.90	225.81 ± 35.68	<0.001^*^
*Vertical*			
Subfovea (μm)	247.55 ± 39.81	236.36 ± 36.86	<0.001^*^
S750 (μm)	236.62 ± 38.97	226.52 ± 38.56	<0.001^*^
S1500 (μm)	243.64 ± 35.24	236.49 ± 34.67	<0.001^*^
I750 (μm)	244.26 ± 39.13	234.41 ± 39.45	<0.001^*^
I1500 (μm)	237.70 ± 40.10	229.50 ± 41.66	<0.001^*^
Mean (μm)	241.96 ± 34.81	232.66 ± 34.61	<0.001^*^
*Emmetropia*			
*Horizontal*			
Subfovea (μm)	315.38 ± 74.97	307.38 ± 69.39	0.010^*^
N750 (μm)	285.76 ± 6.09	284.33 ± 68.49	0.345
N1500 (μm)	243.38 ± 8.46	243.77 ± 74.43	0.916
T750 (μm)	320.12 ± 69.63	325.74 ± 74.45	0.140
T1500 (μm)	325.67 ± 72.44	318.68 ± 68.63	0.277
Mean (μm)	298.06 ± 66.38	295.98 ± 67.26	0.480
*Vertical*			
Subfovea (μm)	312.63 ± 63.97	304.60 ± 64.41	0.011^*^
S750 (μm)	309.82 ± 59.10	301.59 ± 60.47	0.007^*^
S1500 (μm)	305.84 ± 61.17	304.03 ± 71.10	0.752
I750 (μm)	309.16 ± 66.21	303.14 ± 70.49	0.062
I1500 (μm)	304.00 ± 68.22	300.79 ± 68.44	0.206
Mean (μm)	308.29 ± 61.73	302.83 ± 65.46	0.004^*^
*Hyperopia*			
*Horizontal*			
Subfovea (μm)	365.76 ± 83.35	355.20 ± 77.09	0.013^*^
N750 (μm)	345.96 ± 1.70	335.33 ± 90.51	0.030^*^
N1500 (μm)	323.89 ± 79.27	318.33 ± 76.65	0.278
T750 (μm)	358.23 ± 75.58	355.17 ± 70.31	0.609
T1500 (μm)	351.55 ± 73.81	349.76 ± 69.28	0.691
Mean (μm)	349.08 ± 74.12	342.76 ± 72.69	0.035^*^
*Vertical*			
Subfovea (μm)	361.25 ± 87.68	356.01 ± 78.06	0.372
S750 (μm)	365.75 ± 80.15	359.07 ± 76.71	0.016^*^
S1500 (μm)	369.16 ± 77.93	366.20 ± 74.27	0.480
I750 (μm)	334.89 ± 85.36	338.02 ± 80.26	0.542
I1500 (μm)	329.87 ± 75.25	329.05 ± 71.92	0.851
Mean (μm)	352.18 ± 78.73	349.67 ± 73.78	0.482

GEEs for the comparisons before and after accommodation. N, nasal; T, temporal; S, superior; I, inferior.

* Significance of difference.

Similar to the results of CT, the LA, SA, and TCA in both horizontal and vertical scans showed a significant decrease after accommodation in the myopia group (all *p* < 0.05). In the hyperopia group, only the SA in the vertical scan decreased significantly after accommodation (*p* = 0.005). In the emmetropia group, no significant post-accommodation decreases in LA, SA, and TCA were found (all *p* > 0.05; Table 6 and Figure 4).

**Table 6:** Comparisons of CV before and after accommodation in myopia, emmetropia and hyperopia groups.

	Before reading	After reading	*p*
*Myopia*			
*Horizontal*			
Luminal area (LA, μm^2^)	1,015,977 ± 149,135	1,003,086 ± 149,210	0.007^*^
Stromal area (SA, μm^2^)	542,845 ± 78,751	510,518 ± 73,007	<0.001^*^
Total choroidal area (TCA, μm^2^)	1,558,823 ± 214,840	1,513,605 ± 210,205	<0.001^*^
*Vertical*			
Luminal area (LA, μm^2^)	1,108,748 ± 133,397	1,095,828 ± 139,902	0.040^*^
Stromal area (SA, μm^2^)	592,642 ± 73,458	559,071 ± 84,707	<0.001^*^
Total choroidal area (TCA, μm^2^)	1,701,391 ± 193,186	1,654,899 ± 208,568	<0.001^*^
*Emmetropia*			
*Horizontal*			
Luminal area (LA, μm^2^)	1,160,080 ± 261,866	1,153,327 ± 271,519	0.461
Stromal area (SA, μm^2^)	609,105 ± 147,207	587,883 ± 150,509	0.067
Total choroidal area (TCA, μm^2^)	1,769,185 ± 406,063	1,741,211 ± 414,466	0.109
*Vertical*			
Luminal area (LA, μm^2^)	1,262,953 ± 223,965	1,267,481 ± 252,740	0.771
Stromal area (SA, μm^2^)	669,611 ± 126,311	653,707 ± 145,659	0.209
Total choroidal area (TCA, μm^2^)	1,932,564 ± 340,377	1,921,188 ± 384,664	0.664
*Hyperopia*			
*Horizontal*			
Luminal area (LA, μm^2^)	1,292,542 ± 250,185	1,307,921 ± 251,165	0.078
Stromal area (SA, μm^2^)	689,878 ± 129,682	689,769 ± 142,740	0.992
Total choroidal area (TCA, μm^2^)	1,982,421 ± 368,901	1,997,691 ± 385,914	0.345
*Vertical*			
Luminal area (LA, μm^2^)	1,333,644 ± 220,260	1,326,670 ± 217,448	0.443
Stromal area (SA, μm^2^)	703,104 ± 142,895	682,984 ± 144,735	0.005^*^
Total choroidal area (TCA, μm^2^)	2,036,749 ± 355,880	2,009,654 ± 354,006	0.050

GEEs for the comparisons before and after accommodation.

* Significance of difference.

As shown in Table 7, in the myopia, emmetropia, and hyperopia groups, no significant post-accommodation changes in CLA were found in any of the measurement ranges (all *p* > 0.05; Figure 4).

**Table 7:** Comparisons of choriocapillaris luminal area (CLA) before and after accommodation in myopia, emmetropia and hyperopia groups.

	Before reading	After reading	*p*
*Myopia*			
0-1sum (mm^2^)	0.626 ± 0.036	0.624 ± 0.035	0.436
0-3sum (mm^2^)	5.696 ± 0.354	5.655 ± 0.335	0.170
0-6sum (mm^2^)	22.313 ± 1.334	22.106 ± 1.238	0.068
1-3sum (mm^2^)	5.069 ± 0.319	5.031 ± 0.302	0.151
1-6sum (mm^2^)	21.687 ± 1.299	21.482 ± 1.205	0.064
3-6sum (mm^2^)	16.617 ± 0.990	16.451 ± 0.913	0.050
*Emmetropia*			
0-1sum (mm^2^)	0.611 ± 0.040	0.613 ± 0.038	0.756
0-3sum (mm^2^)	5.587 ± 0.368	5.605 ± 0.382	0.694
0-6sum (mm^2^)	22.095 ± 1.260	22.040 ± 1.265	0.662
1-3sum (mm^2^)	4.976 ± 0.331	4.992 ± 0.346	0.689
1-6sum (mm^2^)	21.483 ± 1.224	21.427 ± 1.230	0.642
3-6sum (mm^2^)	16.507 ± 0.904	16.435 ± 0.894	0.397
*Hyperopia*			
0-1sum (mm^2^)	0.623 ± 0.032	0.621 ± 0.027	0.474
0-3sum (mm^2^)	5.719 ± 0.308	5.711 ± 0.285	0.814
0-6sum (mm^2^)	22.545 ± 1.177	22.413 ± 1.215	0.220
1-3sum (mm^2^)	5.096 ± 0.279	5.091 ± 0.260	0.865
1-6sum(mm^2^)	21.922 ± 1.148	21.793 ± 1.192	0.218
3-6sum (mm^2^)	16.826 ± 0.877	16.702 ± 0.940	0.117

GEEs for the comparisons before and after accommodation.

### The Univariate Linear Regression of Associations of Baseline Choroidal Parameters With Age, Sex, Axial Length

In terms of CT and CV, both of them showed significant associations with AL (all *p* < 0.001), whereas none of them was significantly associated with age or sex (all *p* > 0.05). On the contrary, in terms of CLA, besides 0-1sum CLA, the rest measured CLAs were significantly associated with age (all *p* ≤ 0.001). Similarly, 1-3sum, 1-6sum, and 3-6sum CLAs were significantly associated with sex (all *p* < 0.05). However, all the measured CLAs were not significantly associated with AL (all *p* > 0.05; Table 8).

**Table 8:** The univariate linear regression of associations of baseline choroidal parameters with age, sex, axial length.

	Age (years), *p*	Sex (Male/Female), *p*	Axial length (mm), *p*
*Choroidal thickness*			
Horizontal Subfovea (μm)	0.639	0.549	<0.001^*^
N750 (μm)	0.718	0.314	<0.001^*^
N1500 (μm)	0.770	0.448	<0.001^*^
T750 (μm)	0.718	0.696	<0.001^*^
T1500 (μm)	0.557	0.884	<0.001^*^
Horizontal mean (μm)	0.682	0.538	<0.001^*^
Vertical Subfovea (μm)	0.939	0.401	<0.001^*^
S750 (μm)	0.770	0.137	<0.001^*^
S1500 (μm)	0.763	0.318	<0.001^*^
I750 (μm)	0.930	0.361	<0.001^*^
I1500 (μm)	0.841	0.432	<0.001^*^
Vertical mean (μm)	0.844	0.295	<0.001^*^
*Choroidal vascularity*			
Horizontal LA (μm^2^)	0.196	0.644	<0.001^*^
Horizontal SA (μm^2^)	0.372	0.624	<0.001^*^
Horizontal TCA (μm^2^)	0.245	0.630	<0.001^*^
Vertical LA (μm^2^)	0.337	0.582	<0.001^*^
Vertical SA (μm^2^)	0.460	0.693	<0.001^*^
Vertical TCA (μm^2^)	0.329	0.610	<0.001^*^
*Choriocapillaris luminal area*			
0-1sum (mm^2^)	0.351	0.579	0.299
0-3sum (mm^2^)	<0.001^*^	0.278	0.365
0-6sum (mm^2^)	<0.001^*^	0.159	0.884
1-3sum (mm^2^)	<0.001^*^	0.025^*^	0.410
1-6sum (mm^2^)	<0.001^*^	0.014^*^	0.912
3-6sum (mm^2^)	0.001^*^	0.006^*^	0.942

N, nasal; T, temporal; S, superior; I, inferior; LA, Luminal area; SA, Stromal area; TCA, total choroidal area. The linear regression was performed by GEEs. For the association between choroidal parameters and age, sex has been adjusted. For the association between choroidal parameters and sex, age has been adjusted. For the association between choroidal parameters and axial length, age and sex have been adjusted.

* Significance of difference.

## Discussion

In this study, we measured choroidal parameters before and after accommodation in myopia, emmetropia, and hyperopia. We found that the baseline CT and CV were significantly smaller in the myopia group, whereas CLA showed no significant difference among groups. In addition, we conducted 33-cm near reading with an additional −3 D lens as accommodation intervention and found that the CT and CV of the myopia group decreased significantly, whereas only non-significant choroidal thinning and blood perfusion decrease were observed in the emmetropia and hyperopia groups after accommodation. In terms of CLA, no group showed significant changes after accommodation. The association analysis revealed that CT and CV were significantly associated with AL, whereas partial CLAs were significantly associated with age or sex.

Previous animal studies have reported that CT is significantly thinner in myopic eyes and thicker in hyperopic eyes (Howlett and McFadden, 2008; Lu et al., 2009; Zhang et al., 2019). In cross-sectional studies of adult myopic eyes, a significantly thinner choroid could be observed when compared with emmetropic and hyperopic eyes (Tan and Cheong, 2014; Read et al., 2015). In addition, the subgroup analysis revealed that compared with less myopic eyes, the more myopic eyes had a significantly thinner choroid (Wu et al., 2021b). Longitudinal studies indicated that myopic children or emmetropic children with myopia development showed a trend of choroid thinning, whereas non-myopic children had a time-dependent increase in CT (Fontaine et al., 2017; Jin et al., 2019). These study results were consistent with our present study results, which showed that myopic eyes had the thinnest choroid, whereas hyperopic eyes had the thickest choroid. And because the myopic eyes had the thinnest choroid and longest AL, and hyperopic eyes had the thickest choroid and shortest AL, the association analysis of this study showed that CT was significantly associated with AL.

The choroid is composed of a large-vessel (Haller’s) layer and a small- and medium-vessel (Sattler’s) layer, with the choriocapillaris supplied by Haller’s and Sattler’s layer (Wallman et al., 1995). Thus, the choroid is a vascular tissue, and CT cannot fully represent the choroidal circulation (Wu et al., 2021b). Accordingly, recent choroidal studies have assessed choroidal morphology (CT) and perfusion (CV and CLA) simultaneously using SS-OCT/OCTA and found that the thinning of CT in myopia is associated with decreased choroidal blood perfusion (Zhang et al., 2019; Noce et al., 2020; Wu et al., 2021a,b). In terms of choroidal blood perfusion, the choroidal vessel layer was reported to be thinner in myopic eyes and thicker in hyperopic eyes, indicating a close association between decreased choroidal blood perfusion and myopia degree (Esmaeelpour et al., 2014; Tan and Cheong, 2014; Wu et al., 2021b). Devarajan et al. (2020) found that both medium- and large-vessel choroidal layers became thinner with increasing myopia. In addition, other studies have reported that CV (LA, SA, and TCA) decreased significantly with myopia (Zhang et al., 2019; Wu et al., 2021a,b). In this study, we also found that the CV (LA, SA, and TCA) of myopic eyes was significantly smaller, indicating decreased choroidal blood perfusion in myopia. Similar to CT, the CV was also found to be significantly associated with AL in this study.

Previous study in low myopic eyes has suggested that low myopia was found to be associated with macular choroidal thinning, and this decrease in macular CT was mainly caused by the decrease in the choroidal stromal component (Yazdani et al., 2021). In terms of high myopia, similar conclusions were reached, indicating that compared with control eyes, the high myopic eyes had a significant thinner choroid, which was caused by the reduction in choroidal LA and SA. Moreover, the reduction in SA was greater than the reduction in LA in high myopia (Gupta et al., 2017). These study results (Gupta et al., 2017; Yazdani et al., 2021) were highly consistent with ours, which also indicated that both CT and CV (LA and SA) decreased significantly in myopia, and the reduction in SA was greater than the reduction in LA in myopia.

The choriocapillaris has been reported to decrease in eyes with high myopia (Okabe et al., 1982; Quaranta et al., 1996; Al-Sheikh et al., 2017; Su et al., 2020). However, when comparing the choriocapillaris between the normal control and moderate myopia groups, no significant intergroup difference was observed (Su et al., 2020). Moreover, other studies have also suggested that thinning in the choroid is mainly caused by the losses in Haller’s and Sattler’s layers, but not the choriocapillaris (Alshareef et al., 2017; Zhao et al., 2018). Thus, it seems that only high myopic choroid showed significant changes in the choriocapillaris. In this study, our recruited myopic eyes were mild to moderate (mean SE: –2.21 ± 1.26 D), and our choriocapillaris result was consistent with a previous study (Su et al., 2020), indicating no significant choriocapillaris difference among the mild to moderate myopia, emmetropia, and hyperopia groups. Combined with the results of CV, we hypothesized that during development of myopia, the changes in choroidal circulation might first occur in the vessel layers (Haller’s and Sattler’s layers). When myopia development is limited to moderate intensity, the choriocapillaris remains relatively unchanged. Changes in the choriocapillaris would then appear only when myopia develops further into high myopia. Besides that, we could also observe that most measured CLAs were significantly associated with age, indicating that age might have an effect on the choriocapillaris, and the underlying reason still needs to be investigated in the future.

The extent of choroidal thinning was suggested to be one predictor for the levels of myopia and AL (Li et al., 2014; Deng et al., 2018). Before the pathogenesis of myopia, rapid axial elongation could cause thinning of the CT, which may be related to the decrease in choroidal blood flow. Changes in choroid circulation might further result in ischemia and hypoxia of the sclera, resulting in remodeling and impairment of the sclera. Thus, the thinning sclera would be unable to resist the force of axial elongation, leading to further development and acceleration of myopia (Xiong et al., 2020; Wu et al., 2021b). In addition, ischemia and hypoxia of the sclera may also activate downstream receptor-linked signaling pathways and induce the progression of myopia (Zhou et al., 2021).

In this study, we investigated the effect of accommodation on the choroid and found that only myopic eyes showed significant changes in CT and CV, whereas most CT and CV of emmetropic and hyperopic eyes showed non-significant decreases after accommodation. In addition, accommodation induced no significant changes in CLA in the myopia, emmetropia, and hyperopia groups. Changes in the autonomic nervous system of the eye have been suggested to be a potential factor for choroidal changes during accommodation. During accommodation, the ciliary body receives increased parasympathetic signals. In the meantime, the tissues in the choroid, such as non-vascular smooth muscle (NVSM), can also receive increased parasympathetic signals and subsequently contract, leading to the thinning of the choroid during accommodation (Meriney and Pilar, 1987; Flügel-Koch et al., 1996; Poukens et al., 1998; Woodman-Pieterse et al., 2015).

Woodman et al. (2012) found that during accommodation, the CT of myopic eyes decreased significantly, whereas the CT of emmetropic eyes showed no significant changes, indicating that the accommodation-induced thinning in the choroid was more prominent in myopic eyes than in emmetropic eyes. Consistent with the results of Woodman et al., we also observed that only the myopia group showed a significant decrease in CT and CV after accommodation, whereas only non-significant choroidal thinning and blood perfusion decrease were noted in the emmetropia and hyperopia groups. The different responses of the choroid to accommodation stimuli between myopia and emmetropia/hyperopia may result from impairment of the autonomic nervous system in myopic eyes. As mentioned above, accommodation-induced choroidal thinning could be caused by the contraction of the NVSM, controlled by parasympathetic nerves (Meriney and Pilar, 1987; Flügel-Koch et al., 1996; Poukens et al., 1998; Woodman-Pieterse et al., 2015). In terms of autonomic nerves, previous studies have indicated that in myopia, there was an imbalance between sympathetic and parasympathetic nervous systems (Chen et al., 2003) and the choroidal innervation of myopes may be different from emmetropes (Woodman et al., 2012). Thus, we hypothesized that emmetropia and hyperopia could maintain the choroid as relatively unchanged during accommodation because of their normal autonomic nervous system and choroidal innervation, whereas myopic patients were unable to maintain the choroid, unchanged during accommodation, due to its impaired autonomic nervous system and choroidal innervation. Besides autonomic nerves, the anatomical variations of the ciliary muscle of myopic eyes might also contribute to the different responses of the choroid to accommodation stimuli between myopia and emmetropia/hyperopia. Considering that the tendon of the ciliary muscle has been proven to insert into the anterior portion of the choroid, the force of ciliary muscle contraction during accommodation could be transmitted to the choroid, and further stretch the choroid mechanically (Tamm et al., 1991; Woodman et al., 2012), leading to the decreases in CT and CV. In terms of ciliary muscle, previous studies have suggested that there were differences between myopia and emmetropia. Using ultrasound biomicroscopy and OCT, ciliary muscle was found to be thicker in myopic eyes (Bailey et al., 2008; Muftuoglu et al., 2009). Moreover, the posterior ciliary muscle fiber was found to be thicker in myopic eyes, whereas the apical ciliary muscle fiber was found to be thicker in hyperopic eyes (Pucker et al., 2013). Thus, we speculated that the anatomical variation of ciliary muscle between myopia and emmetropia/hyperopia may result in differences in the contraction strength of ciliary muscle during accommodation. Therefore, the stretch force to choroid caused by the contraction of ciliary muscle during accommodation would also be distinct between myopia and emmetropia/hyperopia, making myopia more prone to choroidal thinning and blood perfusion decreasing after accommodation. Therefore, for myopia, which already had a thinner baseline choroid, accommodation could induce further choroidal thinning and blood perfusion decreasing, leading to further ischemia and hypoxia of the sclera and acceleration of axial growth (Xiong et al., 2020; Wu et al., 2021b; Zhou et al., 2021).

Another possibility for myopic choroidal thinning after accommodation might be the delay in myopic choroidal recovery. Previous study has indicated that in young adults, the choroid becomes thinner during accommodation, and subsequently, the choroid would recover to, or be even thicker than, baseline values (Woodman-Pieterse et al., 2015). In this study, the post-accommodation OCT/OCTA measurement was completed in 30–60 s. Thus, during this period, the emmetropic and hyperopic choroids may have already recovered from accommodation-induced thinning and returned to baseline values. Accordingly, they showed no significant differences compared to the pre-accommodation choroidal values. However, in the myopia group, previous studies have reported that after accommodation, the accommodation regression would be delayed, and this delay may be associated with the impaired autonomic nerves in myopia patients (Mallen et al., 2005; Vasudevan and Ciuffreda, 2008). Accordingly, we hypothesized that the impaired autonomic nerves in myopia might also lead to a delay in post-accommodation myopic choroidal recovery. Thus, when we completed the measurements of post-accommodation myopic choroid in 30–60 s, the myopic choroid may not fully recover from accommodation-induced thinning. The accommodation-induced choroidal thinning may last longer in myopic eyes and lead to the further ischemia and hypoxia of the sclera and acceleration of axial growth of myopia (Xiong et al., 2020; Wu et al., 2021b; Zhou et al., 2021).

This study has certain limitations. First, our study sample size was small, and we could not divide myopic eyes into subgroups (such as mild, moderate, and high myopia) for stratification analysis, especially considering that childhood is the main stage of myopia development (Cumberland et al., 2007; Morgan et al., 2012; Golebiewska et al., 2019), making the incidence of high myopia among children relatively low. Second, this study only recruited children; thus, it is unclear whether similar changes could also be observed in adolescents and adults. Third, due to the limitations of our OCT/OCTA instrument, we were unable to observe real-time changes in the choroid during accommodation.

In conclusion, we found that myopic eyes had thinner choroid and lower choroidal blood perfusion than emmetropic and hyperopic eyes. Moreover, myopic eyes were more prone to the choroidal thinning and blood perfusion decreases after accommodation.

## Data Availability Statement

The raw data supporting the conclusions of this article will be made available by the authors, without undue reservation.

## Ethics Statement

The studies involving human participants were reviewed and approved by ethics committee of Tongji Hospital, Tongji Medical College, Huazhong University of Science and Technology. The patients/participants provided their written informed consent to participate in this study.

## Author Contributions

XC, ML, PW, and YX designed this study. XC, ML, LL, XY, YL, MZ, and JW performed the experiments and collected the data. XC, ML, and YX performed the statistical analysis. XC, ML, LL, and PW wrote the manuscript. All authors contributed to the article and approved the submitted version.

## Funding

This work was supported by the National Natural Science Foundation of China (grant nos. 82000893, 81800821).

## Conflict of Interest

The authors declare that the research was conducted in the absence of any commercial or financial relationships that could be construed as a potential conflict of interest.

## Publisher’s Note

All claims expressed in this article are solely those of the authors and do not necessarily represent those of their affiliated organizations, or those of the publisher, the editors and the reviewers. Any product that may be evaluated in this article, or claim that may be made by its manufacturer, is not guaranteed or endorsed by the publisher.

## References

[ref1] Agrawal R. Guota P. Tan K. A. Cheung C. M. Wong T. Y. Cheng C. Y. (2016). Choroidal vascularity index as a measure of vascular status of the choroid: measurements in healthy eyes from a population-based study. Sci. Rep. 6:21090. doi: 10.1038/srep21090, PMID: 26868048 PMC4751574

[ref2] Alonso-Caneiro D. Read S. A. Collins M. J. (2011). Speckle reduction in optical coherence tomography imaging by affine-motion image registration. J. Biomed. Opt. 16:116027. doi: 10.1117/1.3652713, PMID: 22112132

[ref3] Alshareef R. A. Khuthaila M. K. Januwada M. Goud A. Ferrara D. Chhablani J. (2017). Choroidal vascular analysis in myopic eyes: evidence of foveal medium vessel layer thinning. Int. J. Retina Vitreous 3:28. doi: 10.1186/s40942-017-0081-z, PMID: 28560051 PMC5446694

[ref4] Al-Sheikh M. Phasukkijwatana N. Dolz-Marco R. Rahimi M. Iafe N. A. Freund K. B.. (2017). Quantitative OCT angiography of the retinal microvasculature and the choriocapillaris in myopic eyes. Invest. Ophthalmol. Vis. Sci. 58, 2063–2069. doi: 10.1167/iovs.16-21289, PMID: 28388703

[ref5] Bailey M. D. Sinnott L. T. Mutti D. O. (2008). Ciliary body thickness and refractive error in children. Invest. Ophthalmol. Vis. Sci. 49, 4353–4360. doi: 10.1167/iovs.08-2008, PMID: 18566470 PMC2994597

[ref6] Baird P. N. Saw S. M. Lanca C. Guggenheim J. A. Smith E. L. Zhou X.. (2020). Myopia. Nat. Rev. Dis. Primers 6:99. doi: 10.1038/s41572-020-00231-4 33328468

[ref7] Chen J. C. Schmid K. L. Brown B. (2003). The autonomic control of accommodation and implications for human myopia development: a review. Ophthalmic Physiol. Opt. 23, 401–422. doi: 10.1046/j.1475-1313.2003.00135.x, PMID: 12950887

[ref8] Cumberland P. M. Peckham C. S. Rahi J. S. (2007). Inferring myopia over the lifecourse from uncorrected distance visual acuity in childhood. Br. J. Ophthalmol. 91, 151–153. doi: 10.1136/bjo.2006.102277, PMID: 17020900 PMC1857620

[ref9] Dayan Y. B. Levin A. Morad Y. Grotto I. Ben-David R. Goldberg A.. (2005). The changing prevalence of myopia in young adults: a 13-year series of population-based prevalence surveys. Invest. Ophthalmol. Vis. Sci. 46, 2760–2765. doi: 10.1167/iovs.04-0260, PMID: 16043848

[ref10] Deng J. Li X. Jin J. Zhang B. Zhu J. Zou H.. (2018). Distribution pattern of choroidal thickness at the posterior pole in Chinese children with myopia. Invest. Ophthalmol. Vis. Sci. 59, 1577–1586. doi: 10.1167/iovs.17-22748, PMID: 29625482

[ref11] Devarajan K. Sim R. Chua J. Wong C. W. Matsumura S. Htoon H. M.. (2020). Optical coherence tomography angiography for the assessment of choroidal vasculature in high myopia. Br. J. Ophthalmol. 104, 917–923. doi: 10.1136/bjophthalmol-2019-314769, PMID: 31585963

[ref12] Dolgin E. (2015). The myopia boom. Nature 519, 276–278. doi: 10.1038/519276a, PMID: 25788077

[ref13] Esmaeelpour M. Kajic V. Zabihian B. Othara R. Ansari-Shahrezaei S. Kellner L.. (2014). Choroidal Haller’s and Sattler’s layer thickness measurement using 3-dimensional 1060-nm optical coherence tomography. PLoS One 9:e99690. doi: 10.1371/journal.pone.0099690, PMID: 24911446 PMC4050051

[ref14] Flügel-Koch C. May C. A. Lutjen-Drecoll E. (1996). Presence of a contractile cell network in the human choroid. Ophthalmologica 210, 296–302. doi: 10.1159/000310728, PMID: 8878213

[ref15] Fontaine M. Gaucher D. Sauer A. Speeg-Schatz C. (2017). Choroidal thickness and ametropia in children: a longitudinal study. Eur. J. Ophthalmol. 27, 730–734. doi: 10.5301/ejo.5000965, PMID: 28604984

[ref16] Ghosh A. Collins M. J. Read S. A. Davis B. A. Chatterjee P. (2014). Axial elongation associated with biomechanical factors during near work. Optom. Vis. Sci. 91, 322–329. doi: 10.1097/OPX.0000000000000166, PMID: 24413276

[ref17] Golebiewska J. Biala-Gosek K. Czeszyk A. Hautz W. (2019). Optical coherence tomography angiography of superficial retinal vessel density and foveal avascular zone in myopic children. PLoS One 14:e0219785. doi: 10.1371/journal.pone.0219785, PMID: 31318910 PMC6639003

[ref18] Goss D. A. (2000). Nearwork and myopia. Lancet 356, 1456–1457. doi: 10.1016/S0140-6736(00)02864-6 11081523

[ref19] Gupta P. Thakku S. G. Saw S. M. Tan M. Lim E. Tan M.. (2017). Characterization of choroidal morphologic and vascular features in young men with high myopia using spectral-domain optical coherence tomography. Am J. Ophthalmol. 177, 27–33. doi: 10.1016/j.ajo.2017.02.001, PMID: 28209502

[ref20] Holden B. A. Fricke T. R. Wilson D. A. Jong M. Naidoo K. S. Sankaridurg P.. (2016). Global prevalence of myopia and high myopia and temporal trends from 2000 through 2050. Ophthalmology 123, 1036–1042. doi: 10.1016/j.ophtha.2016.01.006, PMID: 26875007

[ref21] Howlett M. H. McFadden S. A. (2008). Spectacle lens compensation in the pigmented Guinea pig. Vis. Res. 49, 219–227. doi: 10.1016/j.visres.2008.10.008, PMID: 18992765

[ref22] Hung L. F. Wallman J. Smith E. L. (2000). Vision-dependent changes in the choroidal thickness of macaque monkeys. Invest. Ophthalmol. Vis. Sci. 41, 1259–1269. PMID: 10798639

[ref23] Ip J. M. Saw S. M. Rose K. A. Morgan I. G. Kifley A. Wang J. J.. (2008). Role of near work in myopia: findings in a sample of Australian school children. Invest. Ophthalmol. Vis. Sci. 49, 2903–2910. doi: 10.1167/iovs.07-0804 18579757

[ref24] Jin P. Zou H. Xu X. Chang T. C. Zhu J. Deng J.. (2019). Longitudinal changes in choroidal and retinal thicknesses in children with myopic shift. Retina 39, 1091–1099. doi: 10.1097/IAE.0000000000002090, PMID: 29517579 PMC6553975

[ref25] Lee K. Park J. H. Park Y. G. Park Y. H. (2020). Analysis of choroidal thickness and vascularity in patients with unilateral polypoidal choroidal vasculopathy. Graefes Arch. Clin. Exp. Ophthalmol. 258, 1157–1164. doi: 10.1007/s00417-020-04620-z, PMID: 32037487

[ref26] Li M. Guo J. M. Xu X. L. Wang J. M. (2017a). Diurnal macular choroidal area fluctuation in normal and primary open angle glaucoma groups. Int. J. Ophthalmol. 10, 1233–1238. doi: 10.18240/ijo.2017.08.08, PMID: 28861348 PMC5554841

[ref27] Li X. Q. Munkholm A. For the Copenhagen Child Cohort 2000 Study Group Larsen M. Munch L. C. (2014). Choroidal thickness in relation to birth parameters in 11- to 12-year-old children: the Copenhagen child cohort 2000 eye study. Invest. Ophthalmol. Vis. Sci. 56, 617–624. doi: 10.1167/iovs.14-15016, PMID: 25358736

[ref28] Li M. Song Y. Zhao Y. Yan X. Zhang H. (2018). Influence of exercise on the structure of the anterior chamber of the eye. Acta Ophthalmol. 96, e247–e253. doi: 10.1111/aos.13564, PMID: 29068522 PMC5836894

[ref29] Li M. Yan X. Q. Song Y. W. Guo J. M. Zhang H. (2017b). Choroidal area assessment in various fundus sectors of patients at different stages of primary open-angle glaucoma by using enhanced depth imaging optical coherence tomography. Medicine (Baltimore) 96:e6293. doi: 10.1097/MD.0000000000006293, PMID: 28272255 PMC5348203

[ref30] Lin H. Long E. Ding X. Diao H. Chen Z. Liu R.. (2018). Prediction of myopia development among Chinese school-aged children using refraction data from electronic medical records: a retrospective, multicentre machine learning study. PLoS Med. 15:e1002674. doi: 10.1371/journal.pmed.1002674, PMID: 30399150 PMC6219762

[ref31] Lu F. Zhou X. Jiang L. Fu Y. Lai X. Xie R.. (2009). Axial myopia induced by hyperopic defocus in Guinea pigs: a detailed assessment on susceptibility and recovery. Exp. Eye Res. 89, 101–108. doi: 10.1016/j.exer.2009.02.019, PMID: 19268468

[ref32] Mallen E. A. Gilmartin B. Wolffsohn J. S. (2005). Sympathetic innervation of ciliary muscle and oculomotor function in emmetropic and myopic young adults. Vis. Res. 45, 1641–1651. doi: 10.1016/j.visres.2004.11.022, PMID: 15792840

[ref33] Meriney S. D. Pilar G. (1987). Cholinergic innervation of the smooth muscle cells in the choroid coat of the chick eye and its development. J. Neurosci. 7, 3827–3839. doi: 10.1523/JNEUROSCI.07-12-03827.1987, PMID: 3694256 PMC6569109

[ref34] Mo J. Duan A. Chan S. Wang X. Wei W. (2017). Vascular flow density in pathological myopia: an optical coherence tomography angiography study. BMJ Open 7:e013571. doi: 10.1136/bmjopen-2016-013571, PMID: 28159853 PMC5294002

[ref35] Morgan I. G. Ohno-Matsui K. Saw S. (2012). Myopia. Lancet 379, 1739–1748. doi: 10.1016/S0140-6736(12)60272-4 22559900

[ref36] Muftuoglu O. Hosal B. M. Zilelioglu G. (2009). Ciliary body thickness in unilateral high axial myopia. Eye 23, 1176–1181. doi: 10.1038/eye.2008.178, PMID: 18551140

[ref37] Noce C. D. Vagge A. Nicolo M. Traverso C. E. (2020). Evaluation of choroidal thickness and choroidal vascular blood flow in patients with thyroid-associated orbitopathy (TAO) using SD-OCT and Angio-OCT. Graefes Arch. Clin. Exp. Ophthalmol. 258, 1103–1107. doi: 10.1007/s00417-020-04616-9, PMID: 32025782

[ref38] Okabe S. Matsuo N. Okamoto S. Kataoka H. (1982). Electron microscopic studies on retinochoroidal atrophy in the human eye. Acta Med. Okayama 36, 11–21. doi: 10.18926/AMO/30704, PMID: 7064730

[ref39] Poukens V. Glasgow B. J. Demer J. L. (1998). Nonvascular contractile cells in sclera and choroid of humans and monkeys. Invest. Ophthalmol. Vis. Sci. 39, 1765–1774. PMID: 9727398

[ref40] Pucker A. Sinnott L. Kao C. Bailey M. D. (2013). Region-specific relationships between refractive error and ciliary muscle thickness in children. Invest. Ophthalmol. Vis. Sci. 54, 4710–4716. doi: 10.1167/iovs.13-11658, PMID: 23761093 PMC3711613

[ref41] Quaranta M. Arnold J. Coscas G. Francais C. Quentel G. Kuhn D.. (1996). Indocyanine green angiographic features of pathologic myopia. Am. J. Ophthalmol. 122, 663–671. doi: 10.1016/s0002-9394(14)70484-2, PMID: 8909205

[ref42] Read S. A. Alonso-Caneiro D. Vincent S. J. Collins M. J. (2015). Longitudinal changes in choroidal thickness and eye growth in childhood. Invest. Ophthalmol. Vis. Sci. 56, 3103–3112. doi: 10.1167/iovs.15-16446, PMID: 26024094

[ref43] Read S. A. Collins M. J. Vincent S. J. Alonso-Caneiro D. (2013). Choroidal thickness in myopic and nonmyopic children assessed with enhanced depth imaging optical coherence tomography. Invest. Ophthalmol. Vis. Sci. 54, 7578–7586. doi: 10.1167/iovs.13-12772, PMID: 24176903

[ref44] Spillmann L. (2020). Stopping the rise of myopia in Asia. Graefes Arch. Clin. Exp. Ophthalmol. 258, 943–959. doi: 10.1007/s00417-019-04555-0, PMID: 31873785

[ref45] Su L. Ji Y. S. Tong N. Sarraf D. He X. Sun X.. (2020). Quantitative assessment of the retinal microvasculature and choriocapillaris in myopic patients using swept-source optical coherence tomography angiography. Graefes Arch. Clin. Exp. Ophthalmol. 258, 1173–1180. doi: 10.1007/s00417-020-04639-2, PMID: 32144487

[ref46] Tamm E. Lütjen-Drecoll E. Junqkunz W. Rohen J. (1991). Posterior attachment of ciliary muscle in young, accommodating old, presbyopic monkeys. Invest. Ophthalmol. Vis. Sci. 32, 1678–1692. PMID: 2016145

[ref47] Tan K. A. Agrawal R. (2015). Luminal and stromal areas of choroid determined by Binarization method of optical coherence tomographic images. Am J. Ophthalmol. 160:394. doi: 10.1016/j.ajo.2015.04.039, PMID: 26187882

[ref48] Tan C. S. Cheong K. X. (2014). Macular choroidal thicknesses in healthy adults–relationship with ocular and demographic factors. Invest. Ophthalmol. Vis. Sci. 55, 6452–6458. doi: 10.1167/iovs.13-13771, PMID: 25228543

[ref49] Tan C. S. Ouyang Y. Ruiz H. Sadda S. R. (2012). Diurnal variation of choroidal thickness in normal, healthy subjects measured by spectral domain optical coherence tomography. Invest. Ophthalmol. Vis. Sci. 53, 261–266. doi: 10.1167/iovs.11-8782, PMID: 22167095

[ref50] Vasudevan B. Ciuffreda K. J. (2008). Additivity of near work-induced transient myopia and its decay characteristics in different refractive groups. Invest. Ophthalmol. Vis. Sci. 49, 836–841. doi: 10.1167/iovs.07-0197, PMID: 18235035

[ref51] Verhoeven V. J. Wong K. T. Buitendijk G. H. Hofman A. Vingerling J. R. Klaver C. C. (2015). Visual consequences of refractive errors in the general population. Ophthalmology 122, 101–109. doi: 10.1016/j.ophtha.2014.07.030, PMID: 25208857

[ref52] Wallman J. Wildsoet C. Xu A. Gottlieb M. D. Nickla D. L. Marran L.. (1995). Moving the retina: choroidal modulation of refractive state. Vis. Res. 35, 37–50. doi: 10.1016/0042-6989(94)e0049-q, PMID: 7839608

[ref53] Wang S. K. Guo Y. Liao C. Chen Y. Su G. Zhang G.. (2018). Incidence of and factors associated with myopia and high myopia in Chinese children, based on refraction without cycloplegia. JAMA. Ophthalmol. 136, 1017–1024. doi: 10.1001/jamaophthalmol.2018.2658, PMID: 29978185 PMC6142978

[ref54] Wei W. B. Xu L. Jonas J. B. Shao L. Du K. F. Wang S.. (2013). Subfoveal choroidal thickness: the Beijing eye study. Ophthalmology 120, 175–180. doi: 10.1016/j.ophtha.2012.07.048 23009895

[ref55] Wildsoet C. Wallman J. (1995). Choroidal and scleral mechanisms of compensation for spectacle lenses in chicks. Vis. Res. 35, 1175–1194. doi: 10.1016/0042-6989(94)00233-c, PMID: 7610579

[ref56] Williams K. M. Werhoeven V. J. Cumberland P. Bertelsen G. Wolfram C. Buitendijk G. H.. (2015). Prevalence of refractive error in Europe: the European eye epidemiology (E(3)) consortium. Eur. J. Epidemiol. 30, 305–315. doi: 10.1007/s10654-015-0010-0, PMID: 25784363 PMC4385146

[ref57] Woodman E. C. Read S. A. Collins M. J. (2012). Axial length and choroidal thickness changes accompanying prolonged accommodation in myopes and emmetropes. Vis. Res. 72, 34–41. doi: 10.1016/j.visres.2012.09.009, PMID: 23017772

[ref58] Woodman E. C. Read S. A. Collins M. J. Hegarty K. J. Perddle S. B. Smith J. M.. (2011). Axial elongation following prolonged near work in myopes and emmetropes. Br. J. Ophthalmol. 95, 652–656. doi: 10.1136/bjo.2010.180323, PMID: 20829316

[ref59] Woodman-Pieterse E. C. Read S. A. Collins M. J. Alonso-Caneiro D. (2015). Regional changes in choroidal thickness associated with accommodation. Invest. Ophthalmol. Vis. Sci. 56, 6414–6422. doi: 10.1167/iovs.15-17102, PMID: 26444722

[ref60] Wu H. Xie Z. Wang P. Liu M. Wang Y. Zhu J.. (2021a). Differences in retinal and choroidal vasculature and perfusion related to axial length in pediatric anisomyopes. Invest. Ophthalmol. Vis. Sci. 62:40. doi: 10.1167/iovs.62.9.40, PMID: 34319397 PMC8322721

[ref61] Wu H. Zhang G. Shen M. Xu R. Wang P. Guan Z.. (2021b). Assessment of choroidal vascularity and choriocapillaris blood perfusion in anisomyopic adults by SS-OCT/OCTA. Invest. Ophthalmol. Vis. Sci. 62:8. doi: 10.1167/iovs.62.1.8, PMID: 33393974 PMC7797932

[ref62] Xiong S. He X. Zhang B. Deng J. Wang J. Lv M.. (2020). Changes in choroidal thickness varied by age and refraction in children and adolescents: a 1-year longitudinal study. Am J. Ophthalmol. 213, 46–56. doi: 10.1016/j.ajo.2020.01.003, PMID: 31945330

[ref63] Yazdani N. Ehsaei A. Hoseini-Yazdi H. Shoeibi N. Alonso-Caneiro D. Collins M. J. (2021). Wide-field choroidal thickness and vascularity index in myopes and emmetropes. Ophthalmic Physiol. Opt. 41, 1308–1319. doi: 10.1111/opo.12875, PMID: 34487376

[ref64] Yoon H. J. Moon H. S. Sung M. S. Park S. W. Heo H. (2021). Effects of prolonged use of virtual reality smartphone-based head-mounted display on visual parameters: a randomised controlled trial. Sci. Rep. 11:15382. doi: 10.1038/s41598-021-94680-w, PMID: 34321504 PMC8319184

[ref65] Zhang S. Zhang G. Zhou X. Xu R. Wang S. Guan Z.. (2019). Changes in choroidal thickness and choroidal blood perfusion in Guinea pig myopia. Invest. Ophthalmol. Vis. Sci. 60, 3074–3083. doi: 10.1167/iovs.18-26397, PMID: 31319419

[ref66] Zhang Q. Zheng F. Motulsky E. H. Gregori G. Chu Z. Chen C. L.. (2018). A novel strategy for quantifying choriocapillaris flow voids using swept-source OCT angiography. Invest. Ophthalmol. Vis. Sci. 59, 203–211. doi: 10.1167/iovs.17-22953, PMID: 29340648 PMC5770182

[ref67] Zhao J. Wang Y. X. Zhang Q. Wei W. B. Xu L. Jonas J. B. (2018). Macular choroidal small-vessel layer, Sattler’s layer and Haller’s layer thicknesses: the Beijing Eye Study. Sci. Rep. 8:4411. doi: 10.1038/s41598-018-22745-4, PMID: 29535365 PMC5849687

[ref68] Zhou X. Ye C. Wang X. Zhou W. Reinach P. Qu J. (2021). Choroidal blood perfusion as a potential “rapid predictive index” for myopia development and progression. Eye Vis. 8:1. doi: 10.1186/s40662-020-00224-0, PMID: 33397473 PMC7780679

[ref69] Zhu X. Park T. W. Winawer J. Wallman J. (2005). In a matter of minutes, the eye can know which way to grow. Invest. Ophthalmol. Vis. Sci. 46, 2238–2241. doi: 10.1167/iovs.04-0956 15980206

